# The Microbial Ecosystem Distinguishes Chronically Diseased Tissue from Adjacent Tissue in the Sigmoid Colon of Chronic, Recurrent Diverticulitis Patients

**DOI:** 10.1038/s41598-017-06787-8

**Published:** 2017-08-16

**Authors:** Kathleen M. Schieffer, Kate Sabey, Justin R. Wright, David R. Toole, Rebecca Drucker, Vasily Tokarev, Leonard R. Harris, Sue Deiling, Melanie A. Eshelman, John P. Hegarty, Gregory S. Yochum, Walter A. Koltun, Regina Lamendella, David B. Stewart

**Affiliations:** 1 Division of Colon and Rectal Surgery, Department of Surgery, The Pennsylvania State University, College of Medicine, Hershey, PA USA; 2 Department of Biology, Juniata College, Huntingdon, PA USA; 3 Wright Labs LLC., Huntingdon, PA USA; 4 Department of Biochemistry & Molecular Biology, The Pennsylvania State University, College of Medicine, Hershey, PA USA

## Abstract

Diverticular disease is commonly associated with the older population in the United States. As individual’s age, diverticulae, or herniation of the mucosa through the colonic wall, develop. In 10–25% of individuals, the diverticulae become inflamed, resulting in diverticulitis. The gut ecosystem relies on the interaction of bacteria and fungi to maintain homeostasis. Although bacterial dysbiosis has been implicated in the pathogenesis of diverticulitis, associations between the microbial ecosystem and diverticulitis remain largely unstudied. This study investigated how the cooperative network of bacteria and fungi differ between a diseased area of the sigmoid colon chronically affected by diverticulitis and adjacent non-affected tissue. To identify mucosa-associated microbes, bacterial 16S rRNA and fungal ITS sequencing were performed on chronically diseased sigmoid colon tissue (DT) and adjacent tissue (AT) from the same colonic segment. We found that *Pseudomonas* and *Basidiomycota* OTUs were associated with AT while *Microbacteriaceae* and *Ascomycota* were enriched in DT. Bipartite co-occurrence networks were constructed for each tissue type. The DT and AT networks were distinct for each tissue type, with no microbial relationships maintained after intersection merge of the groups. Our findings indicate that the microbial ecosystem distinguishes chronically diseased tissue from adjacent tissue.

## Introduction

The gut comprises an ecological network of microbes, which together are a major determinant of the colonic microenvironment. As a part of the colonic ecosystem, bacteria (microbiome) and fungi (mycobiome) cohabitate, potentially influencing one another’s pathogenicity, nutritional states, symbiosis, and development of community structures^1^. In states of good health, gut microbes synergistically coexist with the host to the benefit of each, providing host nutrition, maintaining homeostasis, and preventing disease^1^. Disruption of the gut ecosystem can result in pathogenic consequences. In particular, bacterial dysbiosis has been associated with various systemic disease states, including obesity^2^, inflammatory bowel disease (IBD)^3^, and *Clostridium difficile* infection^4^. More recently, the involvement of fungal communities associated with disease, such as in IBD^5, 6^, has gained attention. Comprehensive analysis of the microbial ecosystem may help explain the development of these various disease states.

Diverticular disease affects about 2.5 million people in the United States each year^7^. It is commonly a disease associated with age, being found in 40% of people over the age of 60 with an increasing incidence with advancing age^8^. Diverticular disease includes a spectrum of changes to the gut that begin with diverticulosis, or the herniation of mucosa and muscularis mucosa through the wall of the colon, usually in areas where mural blood vessels pierce the muscle layer of the bowel wall^8, 9^. In Western countries, 75–90% of diverticulosis occurs in the sigmoid colon^10–12^. In approximately 10–25% of diverticulosis patients, the diverticulae becomes inflamed, leading to diverticulitis^8^. Diverticulitis is empirically treated with broad spectrum antibiotics, suggesting that bacteria may contribute to its pathogenesis. In some cases, individuals with chronic, recurrent episodes of diverticulitis may require surgical resection of the sigmoid colon.

There is limited data suggesting that bacterial dysbiosis may be an important determinant in the pathogenesis of diverticulitis^13, 14^. However, research on this topic is challenged by the difficulty in identifying an appropriate control group since, for reasons that are still unclear, many persist with asymptomatic diverticulosis. Additionally, the microbial ecosystem relies on relationships between bacteria and fungi, so the exclusion of fungal organisms in prior studies on this subject omits a potential key causal factor in this disease. In the present study, both bacteria and fungi were investigated separately as well as through transkingdom interactions to determine whether microbial differences in tissue chronically affected by diverticulitis (DT) versus adjacent non-affected tissue (AT) can distinguish these two tissue types.

Using 16S rRNA and internal transcribed spacer (ITS) gene sequencing, we assigned bacterial and fungal sequencing reads to operational taxonomic units (OTUs) and analyzed how the microbial ecosystem differed between patient-matched DT from the sigmoid colon and AT from the same colonic segment. Underlying the high similarity of commensal organisms, we hypothesized that a distinctive subset of mucosa-associated pathogenic microbes would be associated with DT relative to AT, and that the microbial network of bacteria and fungi would differ between the two tissue types. Inferred metagenomic analyses were performed to evaluate the predictive metagenomes of the microbial communities associated with DT and AT as further explanation of an association between the microbiome and diverticulitis. Our study describes distinct microbial ecological networks which distinguish DT and AT, and these data suggest that a dysbiotic network of microbes is associated with the mucosa of chronically diseased sigmoid colon.

## Results

### Unique bacterial taxa colonize chronically diseased and adjacent tissue

**Figure 1:**
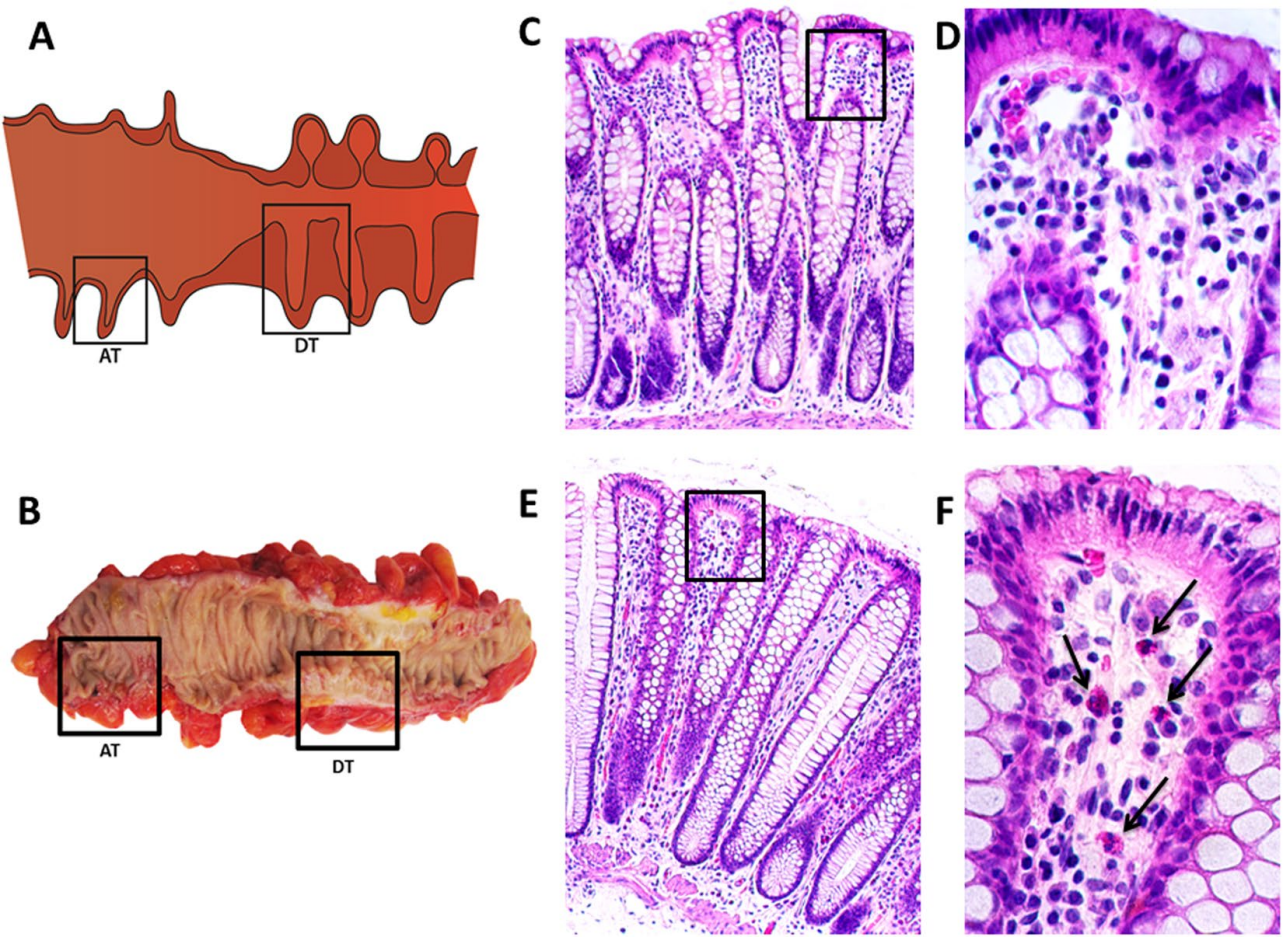
Evaluation of chronically diseased tissue and adjacent tissue obtained from the sigmoid colon of chronic, recurrent diverticulitis patients. **(A**) Illustrated representation of the sigmoid colon with areas obtained for analysis shown in boxes. Chronically diseased tissue (DT) is an area of thickened bowel wall. Adjacent tissue (AT) is an area of normal bowel wall thickness. Diverticulae may be present in either tissue section. **(B**) Sigmoid colon taken from a patient immediately after resection and representative tissue sections shown in boxes. **(C**) Representative H&E stained section from AT shows minimal mucosal neutrophilic inflammation. **(D**) Higher magnification of the boxed area of **(C)** showing negligible mucosal inflammation. **(E**) Representative H&E stained section from DT with minimal mucosal inflammation demonstrated by the presence of few mucosal neutrophils within the lamina propria. **(F**) Higher magnification of the boxed area of **(E**) with neutrophils marked by an arrow.

Prior to assessing the mucosa-associated bacterial community structure, we evaluated the cellular architecture and inflammatory cell infiltrate present in DT and AT. Hematoxylin and eosin stained tissue sections were examined from nine patients whose clinical demographics are described in Table 1. DT is an area chronically affected by diverticulitis that demonstrated a thickened bowel wall, while AT is obtained from an area adjacent to DT and is unaffected by disease (Fig. 1A,B). The cellular architecture for both DT and AT was normal-appearing, with an intact epithelium and crypts. AT showed negligible mucosal neutrophilic inflammation (Fig. 1C,D), whereas DT also showed minimal inflammation but demonstrated increased numbers of neutrophils within the lamina propria (Fig. 1E,F).

**Table 1:** Demographics and clinical information.

	Diverticulitis cohort (n = 9)
Sex (male/female)	5/4
Age (years) at surgery, mean ± SD (range)	60.9 ± 14.1 (43–77)
Body mass index (kg/m^2^) mean ± SD (range)	25.0 ± 2.8 (20–28)
Episodes of diverticulitis prior to surgery	
1–2 episodes	4
3–4 episodes	2
≥5 episodes	3
Antibiotics usage within 3 months prior to surgery	
Metronidazole and Neomycin^1^	9
Metronidazole and Ciprofloxacin	2
Amoxicillin/Clavulanic Acid	1
Doxycycline and Cefalexin	1

SD, standard deviation. ^1^Received day prior to surgery as part of mechanical bowel prep.

**Figure 2:**
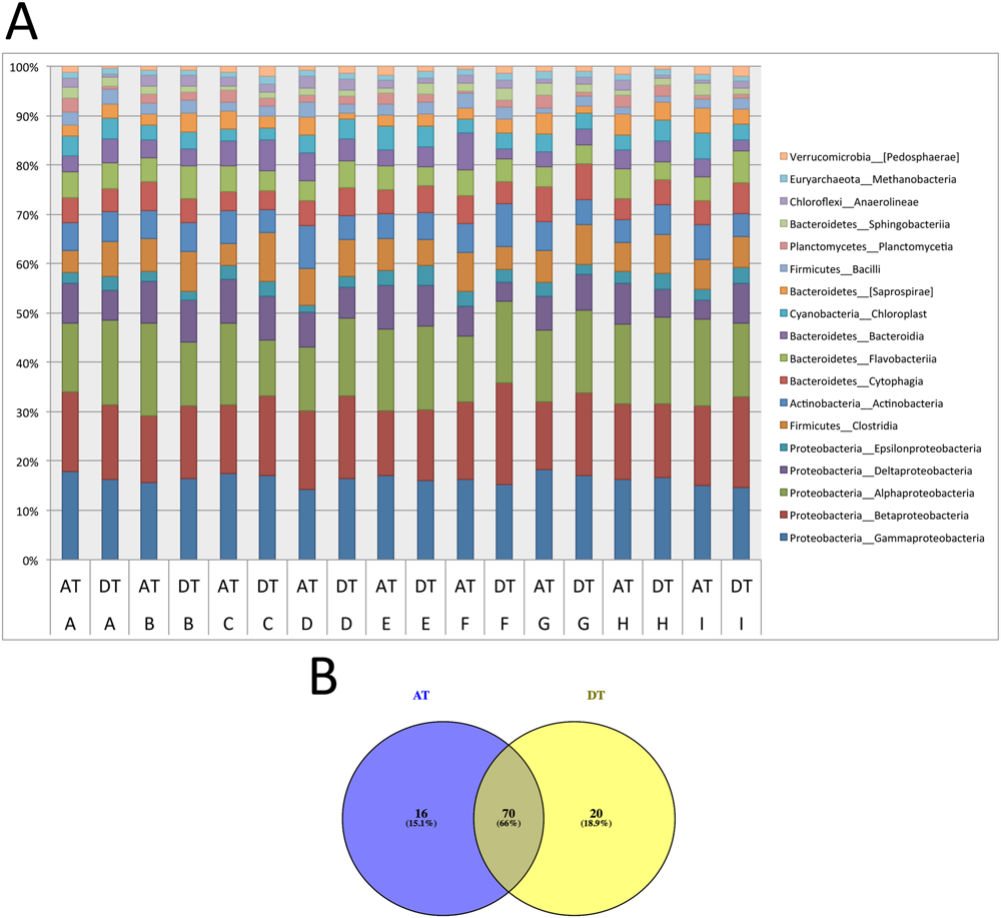
Bacterial alpha and beta diversity of chronically diseased tissue and adjacent tissue. **(A)** Bacterial community composition profiles illustrate abundances of prevalent taxonomic classes. All taxa unassigned at the kingdom level were removed. Patients are arbitrarily labeled A through I with chronically diseased tissue (DT) and adjacent tissue (AT) indicated. **(B**) Venn diagram of core microbiome (including OTUs found in ≥80% of the samples) presents the number of OTUs that are shared (66.0%) and unique between chronically diseased tissue (DT) (18.9%) and adjacent tissue (AT) (15.1%).

We then performed 16S rRNA gene sequencing to analyze the microbiome of patient-matched DT and AT and found that bacterial communities in both tissue types were dominated by proteobacterial taxa (Fig. 2A). Alpha diversity measures did not reveal differing trends between DT and AT, likely due to the use of patient-matched samples resulting in high similarity of commensal organisms (Supplemental Fig. S1A). Overall clustering based on weighted UniFrac distance was not significant when comparing tissue type (ANOSIM test *P* = 0.521) (Supplemental Fig. S1B) or patient identification (ANOSIM test *P* = 0.110) (Supplemental Fig. S1C).

We next analyzed the core microbiome, which typically refers to the population of bacteria comprising two or more habitats^15^. In our study, the core microbiome was defined as common bacteria with OTUs present in ≥80% of all samples. This analysis revealed that subsets of bacterial taxa were distinct between DT and AT (Fig. 2B). Although a majority of OTUs (66.0%) were shared among the two tissue types, 18.9% and 15.1% of OTUs were specific to DT and AT, respectively (Supplemental Table S2).

**Figure 3:**
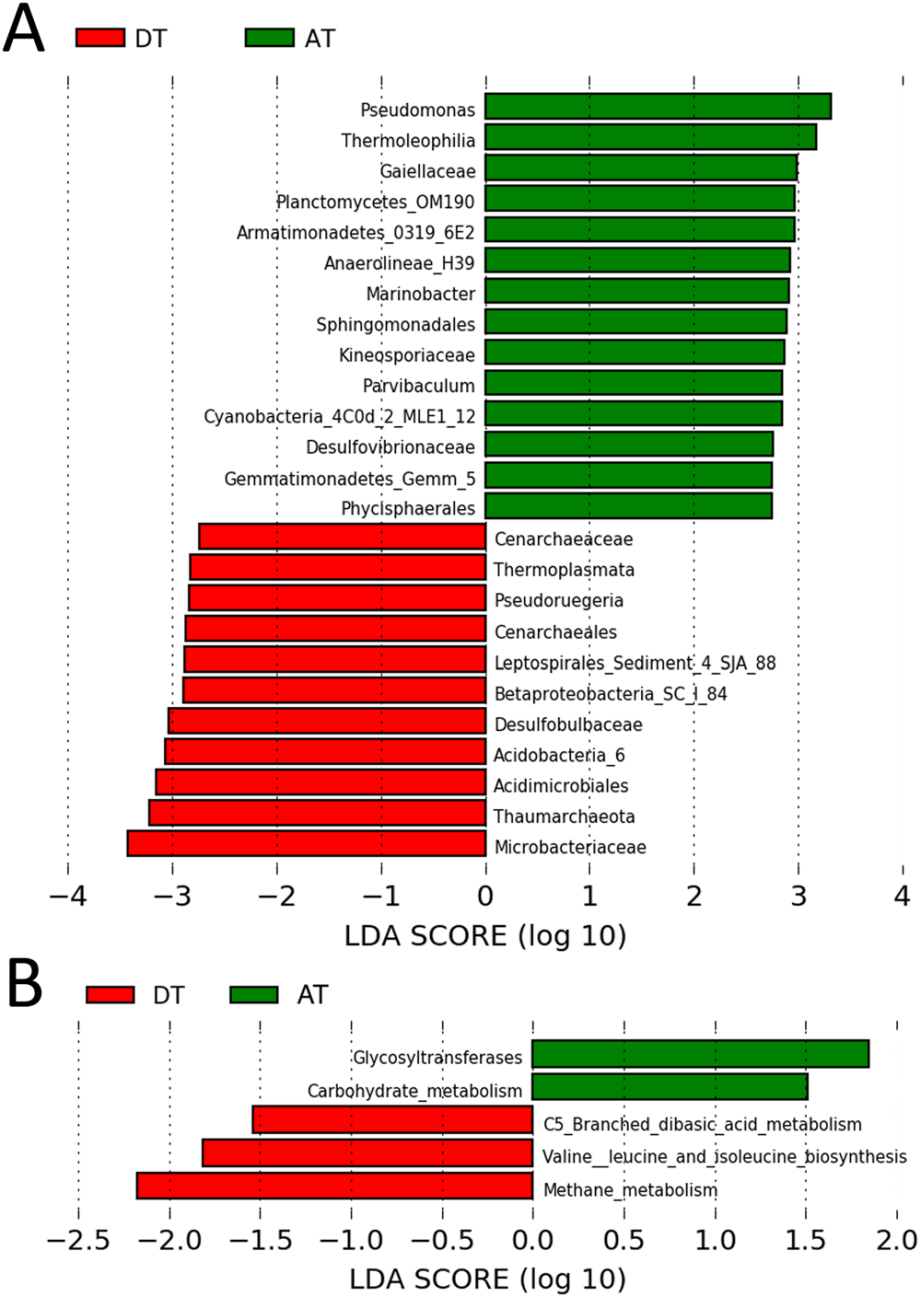
Differential bacterial and archaeal compositions comprise chronically diseased tissue and adjacent tissue. **(A**) LDA Effect Size (LEfSe) plot of taxonomic biomarkers identified within chronically diseased tissue (DT) and adjacent tissue (AT) samples. Red bars are indicative of enrichment within DT samples, whereas green bars are indicative of enrichment within AT samples. A Kruskal-Wallis test was employed at an alpha = 0.05 to identify significantly enriched taxa, whereas a pairwise Wilcoxon rank sum test was utilized to test biological consistency across all subgroups (alpha = 0.05). A linear discriminant analysis (LDA) was calculated to determine effect size and we present all taxa that yielded an LDA score >1.5. A total of 33 taxa (25 being taxonomically identified) were found to be enriched in both sample groupings. **(B**) PICRUSt-inferred metagenomics and LEfSe predicted-function enrichment plots. Functions with an LDA score >1.5 are shown. Inferred molecular function of the bacterial populations identified by 16S rRNA gene sequencing are stratified by tissue type (chronically diseased tissue (DT) and adjacent tissue (AT).

To further define which bacterial taxa were enriched in each tissue type, Linear discriminant analysis Effect Size (LEfSe) analysis was used (Fig. 3A). A total of 33 taxa (25 being taxonomically identified) were enriched in both groups, with a linear discriminant analysis (LDA) score >1.5 (Supplemental Table S3). In general, bacteria within the phyla *Proteobacteria* and *Actinobacteria* were most represented in the LEfSe plot for DT and AT. The OTU *Pseudomonas* (*P* = 0.038) was most predictive of AT while *Microbacteriaceae* (*P* = 0.019) were enriched in DT. We then used Phylogenetic Investigation of Communities by Reconstruction of Unobserved States (PICRUSt) to infer the potential mechanistic function from virtual metagenomics data collected from 16S rRNA sequencing (Fig. 3B). NSTI scores were calculated on all 23 samples included in PICRUSt metagenome prediction analysis and yielded an average score of 0.108 ± 0.013. Five pathways were identified with an LDA score >1.5. AT was associated with enrichment of glycosyltransferase (*P* = 0.002) and carbohydrate metabolism (*P* = 0.024) pathways while DT was correlated with methane metabolism (*P* = 0.047); valine, leucine, and isoleucine biosynthesis (*P* = 0.031); and the C5-branched dibasic acid metabolism pathway (*P* = 0.009) (Supplemental Table S4). In accordance with the observed association between methane metabolism and DT, methanogenic archaea, such as *Thermoplasmata*, were found to be predictive of DT (Fig. 3A).

### Pathogenic fungal species are found in chronically diseased tissue

**Figure 4:**
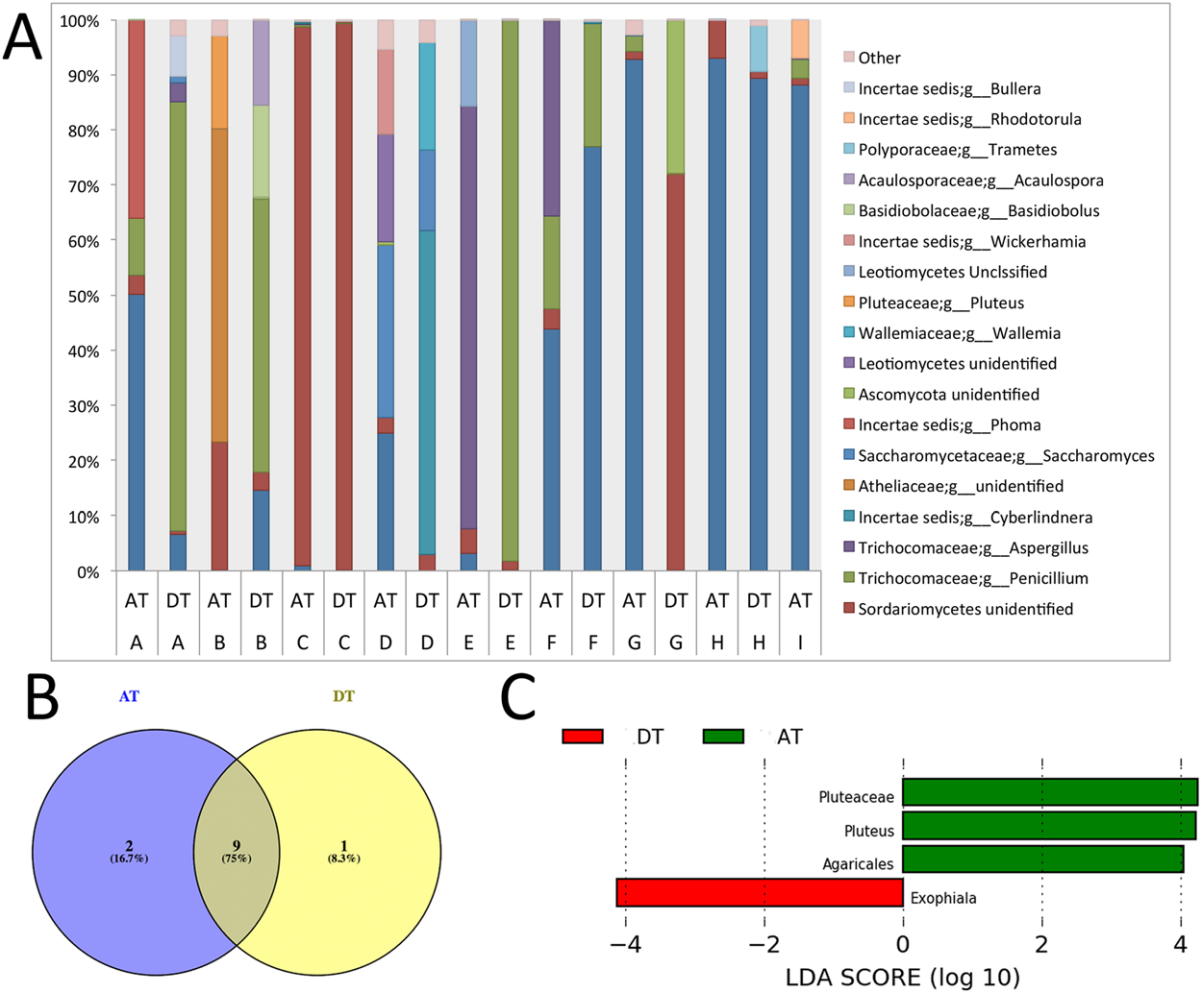
Alpha and beta diversity analysis of fungal communities associated with chronically diseased tissue and adjacent tissue. **(A**) Fungal community composition profiles illustrate abundances of prevalent taxonomic orders and families. All taxa unassigned at the kingdom level were removed. Patients are arbitrarily labelled A-I with chronically diseased tissue (DT) and adjacent tissue (AT) indicated. (**B**) Venn diagram of core mycobiome (including OTUs found in ≥80% of the samples) displays the number of OTUs that are similar (75.0%) and unique between chronically diseased tissue (DT) (8.3%) and adjacent tissue (AT) (16.7%). **(C)** LDA Effect Size (LEfSe) enrichment plots reveal significantly enriched fungi identified within each tissue type (chronically diseased tissue (DT) and adjacent tissue (AT)). Taxa with an LDA score >1.5 are presented.

Fungal organisms also constitute a large part of the intestinal ecosystem and interact with bacteria to influence their community structure, so we next analyzed the mycobiome of DT and AT. At the fungal class level, we detected large abundance profile differences for within-subject variation and between-subject variation among tissue types (Fig. 4A). Similar to the bacterial analysis, alpha diversity measures for fungal communities did not reveal significant differences between DT and AT (Supplemental Fig. S2A). While weighted UniFrac distance analysis showed no significant clustering of overall fungal community structure based on tissue type (ANOSIM test *P* = 0.635) (Supplemental Fig. S2B), analysis by patient identification did reveal significant clustering (ANOSIM test *P* = 0.010) (Supplemental Fig. S2C). Core mycobiome analysis (OTUs present in ≥80% of all samples) revealed that a majority of OTUs (75.0%) were shared between DT and AT (Fig. 4B). Only 8.3% and 16.7% of OTUs were specific to DT and AT, respectively (Supplemental Table S5). Using LEfSe to identify fungal taxonomic biomarkers for DT and AT with an LDA score >1.5, the OTU *Exophiala* found in the division *Ascomycota* was enriched in DT (*P* = 0.037) while three *Basidiomycota* OTUs identified to *Plutaceae* (*P* = 0.039), *Pluteus* (*P* = 0.039), and *Agaricales* (*P* = 0.039) were correlated with AT (Fig. 4C) (Supplemental Table S6). Because examining the mycobiome did not identify sufficient evidence to suggest that fungal organisms alone may be involved in diverticular disease, we analyzed the interactive network of bacterial-fungal relationships.

### Distinct bipartite co-occurrence networks describe chronically diseased tissue and adjacent tissue

**Figure 5:**
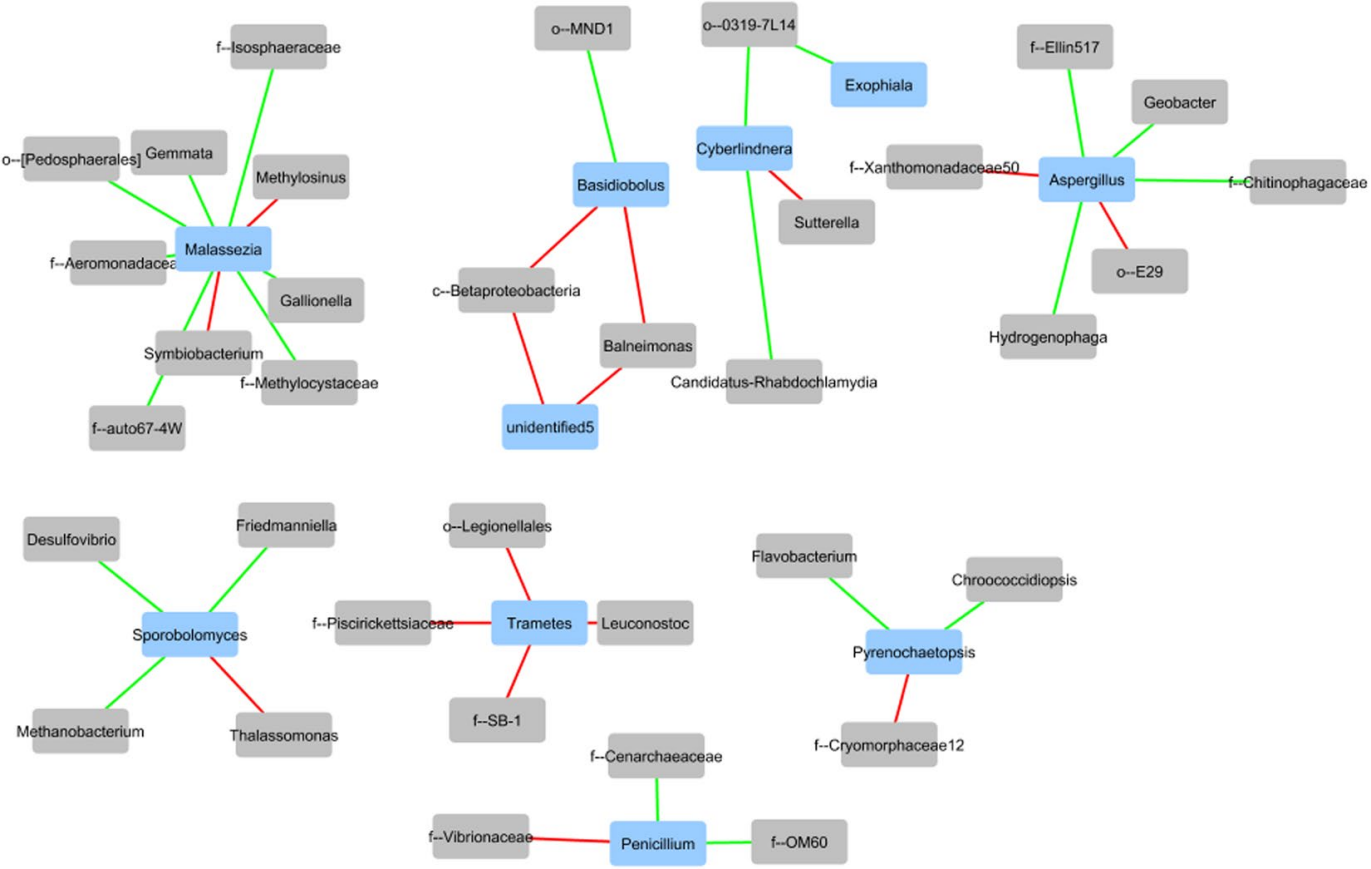
Bipartite co-occurrence network of chronically diseased tissue showing positive (green) and negative (red) correlations between fungal (blue) and bacterial (gray) organisms (Spearman’s rho >0.80). Nodes are labeled to the furthest identified taxonomic level. When merged with the adjacent tissue bipartite co-occurrence network plot, none of the described relationships were maintained.

The gut ecosystem thrives on relationships between microbes to promote a healthy environment and physiological homeostasis, and in turn, disruption of this homeostatic ecosystem may promote disease states^2–6^. To examine how bacteria and fungi co-exist to potentially maintain the ecosystem of DT and AT, bipartite co-occurrence networks were constructed and analyzed. Positive and negative bacterial-fungal relationships were determined for each tissue type identifying co-existence or competitive exclusion, respectively^16^. The bipartite co-occurrence network plot for DT is presented (Fig. 5). As predicted, both positive and negative correlations (Spearman’s rho >0.80) were observed, confirming the presence of an interactive network of microorganisms. Using the intersection merge method to evaluate the bacterial-fungal relationships that are similar and different between DT and AT bipartite networks, we found that each tissue type exhibited differential microbial interactions, suggesting distinct microbial ecologies. For example, enrichment of the OTU *Pseudomonas* was identified as a taxonomic biomarker of AT (Fig. 3A) and this OTU had a positive relationship with the fungal OTU *Aspergillus* in AT; however, in DT, this relationship was not observed. Thus, this analysis identified differences in the microbial ecosystem and bacterial-fungal relationships between DT and AT. Such transkingdom interactions allows for a broad overview of the ecological niche that may play a role in the inflammatory processes seen in areas of chronic disease.

## Discussion

Our multi-faceted approach of analyzing the microbiome, the mycobiome, and the ecological relationship between bacteria and fungi allowed us to glean several potentially important insights into the differences between chronically diseased diverticular tissue and adjacent non-inflamed sigmoid colon. LEfSe analysis found that AT was associated with enrichment of *Pseudomonas* and *Basidiomycota* OTUs while *Microbacteriaceae* and *Ascomycota* were enriched in DT. Unique microbial ecological networks distinguished the two tissue types, with no relationships maintained upon merge of the two bipartite co-occurrence network plots. These data suggest that distinct microbial ecosystems may have a role in the inflammatory process associated with diverticular disease.

In line with our results, a previous study found higher diversity of *Proteobacteria,* with *Pseudomonas* as one of the predictive OTUs for diverticulitis patients compared to IBD and colorectal cancer patients^14^. However, whether this enrichment is associated with non-specific intestinal inflammation^17^ or the diverticular disease process will require further research. *Pseudomonas* is an organism which in healthcare settings is known to have a broad range of behavior, but with a potential for antibiotic resistance^18^. Animal models of intestinal anastomoses have shown that *Pseudomonas* can release collagenases in response to tissue injury^19^. This may have a correlation with diverticular disease, where an initial mechanical insult from biochemical stimuli or stool within the diverticulae may promote the release of degradative enzymes by this opportunistic pathogen.

PICRUSt allowed the inference of mechanistic pathways potentially associated with different bacterial communities. One of the enriched pathways predicted by PICRUSt, was methane metabolism, which is consistent with LEfSe findings that *Thermoplasmata* are predictive of DT, as archaea within this class have been identified as methylamine-degrading, gut methanogens^20–22^. Previous studies similarly found an enrichment of methanogenic bacteria, such as *Methanobrevibacter smithii*, in patients with diverticulosis^23^ and with constipation^24^. A predominance of methanogenic microorganisms may, therefore, be associated with gut motility patterns which lend to the development of diverticulae. It should be emphasized that PICRUSt is a computational method used to analyze virtual metagenomic data, and due to its inferred approach, physiological interpretations of these results should be treated with caution as they require experimental confirmation. Future work should include shotgun metagenomics and meta-transcriptomics sequencing to elucidate the genetic potential and activities within these gut microbial communities.

Beta diversity analyses revealed that overall fungal community structure was more strongly driven by inter-individual variation than tissue type, suggesting that diverticulitis is perhaps not associated with fundamental differences in mycobiome composition, but shifts in a subset of taxa and their accompanying interactivity with the rest of the community. Fungal sequencing data found an enrichment of OTUs associated with *Ascomycota* in DT, while AT was associated with *Basidiomycota* OTUs. A prior study that correlated diet with gut ecology noted an inverse relationship in enrichment between these two taxa, and a correlation between these fungi and certain bacterial OTUs such as *Prevotella* and *Bacteroides* ^25^. How these fungal organisms might potentially contribute to diverticulitis is unknown at this time, although in IBD, a gut disease known to be associated with a chronic intestinal dysbiosis and immune dysregulation, at least one previous study reported an increased *Basidiomycota/Ascomycota* ratio from fecal samples^5^. The variable size of the ITS region along with relatively poor sequencing of reverse reads in our study prevented analysis of paired-end sequence data. However, a previous report indicates that the analysis of high quality single reads provides robust representation of present communities^26^. Nevertheless, how different fungal organisms may contribute to varying bacterial ecologies and host immune responses represents a currently unstudied aspect of diverticulitis as well as most other gut diseases.

Using tissue samples to assess the mucosa-associated microbiome rather than stool limits our study to only patients who require surgery for their disease. Additionally, these patients demonstrate a more severe phenotype by virtue of their need for surgery. All such patients required antibiotics pre-operatively; however, only four patients were on antibiotics in the three months prior to surgery. Antibiotic usage (Table 1) did not influence bacterial species richness (cephalexin *P* = 0.192, neomycin *P* = 0.175, amoxicillin and clavulanic acid *P* = 0.151, and doxycycline *P* = 0.192) or fungal species richness (cephalexin *P* = 0.080, neomycin *P* = 0.162, amoxicillin and clavulanic acid *P* = 0.085, and doxycycline *P* = 0.101). By utilizing tissue, we are able to examine the mucosa-associated population of microbes rather than cataloguing those excreted in the stool. This obtains a more complete analysis of mechanistically-relevant taxa for a disease whose hallmark is changes to the colon wall. Given the shape of diverticulae, we believe that using mucosa-associated organisms is of particular importance. Diverticulae can have narrow necks, limiting communication with the lumen of the colon and helping to create a physically partitioned, distinct microbial environment.

While perhaps at the cost of a larger sample size, another priority in experimental design was to limit the study population to well-matched and carefully selected cohorts of diverticulitis patients. To overcome inter-individual differences, each subject was used as their own control, by assaying AT collected from a macroscopically non-diseased section of sigmoid colon at the time of surgical resection. The resulting small sample size also hindered the ability to incorporate clinical data as a correlate to microbial findings, and larger studies will be necessary in this regard. Consequently, this is the first diverticulitis study to examine the microbiome using a control group that does not harbor a confounding bacterial community structure, such as in IBD patients who harbor an intestinal dysbiosis at baseline^14^.

In summary, we provide a further description of the microbial communities associated with diverticulitis. The inclusion of fungal organisms in microbial analyses of gut diseases has been lacking, and this study represents the first investigation of the mycobiome in diverticulitis. The finding of unique communities of both bacteria and fungi indicate the need to incorporate both kingdoms in future microbiome analyses. Our study additionally demonstrates potential differences between DT and AT in terms of microbial functionality. Future directions in this aim could incorporate matched metagenomics and meta-transcriptomics to glean possible roles of gut microbes in shaping diverticular etiology and progression, as well as host cell transcriptomics to define host-pathogen interactions. Development of a currently unavailable animal model for diverticulitis would also aid in characterizing pertinent microorganisms and potential treatment therapies. In total, examining the interactions between bacterial and fungal species in diverticular disease, evaluating the role anti-fungal agents may have in the treatment of diverticulitis, and exploring microbial metabolic activity, would help to further understand the impact of these organisms on disease pathophysiology.

## Materials and Methods

### Study design and specimen collection

This retrospective cohort study was performed at the Penn State Hershey Medical Center with Institutional Review Board (IRB) approval. Informed consent was obtained from all subjects and all methods were performed in accordance with these guidelines. Between April 2010 and August 2014, chronic, recurrent diverticulitis patients were consented to collect colonic tissue into the Penn State Hershey Colon and Rectal Diseases Biobank at the time of elective sigmoid resection. Surgical specimens were immediately transported from the operating room to the surgical pathology laboratory where Biobank staff obtained several full-thickness sections of tissue. Chronically diseased tissue (DT) was an area of chronic inflammation that demonstrated a thickened bowel wall. Adjacent tissue (AT) sections were taken from an area of sigmoid colon with normal appearing bowel wall thickness, and as far away from DT as possible (Fig. 1A,B). This section comprised our patient-matched control that was not influenced by chronic, recurrent diverticulitis. Tissue was flash-frozen at −80 °C until processing for analysis. Confirmation of diverticulitis was based on preoperative CT scans and surgical pathology. Patients with IBD, cancer, or dysplasia were excluded.

### Analysis of the microbiome

To analyze populations of bacteria associated with the mucosa as opposed to those present in the stool, DNA was extracted from approximately 250 mg of colonic tissue using the Qiagen DNeasy Blood and Tissue Kit (Qiagen, Frederick, MD). DNA was quantified using a Nanodrop 2000 (ThermoFisher Scientific, Waltham, MA), aliquoted, and shipped on dry ice to Dr. Lamendella at Juniata College. DNA concentrations were quantified with the Qubit 2.0 Fluorometer High Sensitivity dsDNA kit (Life Technologies, Carlsbad, CA) according to manufacturer’s instructions. PCR was performed using the Illumina-barcoded 806 R reverse primer and 515 F forward primer as previously described^4^. Pooled PCR products were gel purified using the Qiagen Gel Purification Kit (Qiagen, Frederick, MD), quantified using the Qubit 2.0 Fluorometer (Life Technologies, Carlsbad, CA), and samples were combined in equimolar amounts. Prior to submission for sequencing, libraries were quality checked using the 2100 Bioanalyzer DNA 1000 chip (Agilent Technologies, Santa Clara, CA). Pooled libraries were stored at −20 °C until they were shipped on dry ice to the California State University (North Ridge, CA) for sequencing.

Library pools were size verified using the Fragment Analyzer CE (Advanced Analytical Technologies Inc., Ames, IA) and quantified using the Qubit High Sensitivity dsDNA kit (Life Technologies, Carlsbad, CA). After dilution to a final concentration of 1 nM and addition of a 10% spike of PhiX V3 library as an internal control (Illumina, San Diego CA), pools were denatured for 5 minutes in an equal volume of 0.1 N NaOH then further diluted to 12 pM in Illumina’s HT1 buffer. The denatured and PhiX-spiked 12 pM pool was loaded on an Illumina MiSeq V2 300 cycle kit cassette with 16S rRNA library sequencing primers and set for 150 base, paired-end reads.

Forward and reverse reads were merged using VSEARCH version 1.9.10 with a minimum overlap set to 40 bp^26^. Using USEARCHv7, paired sequences were quality filtered at a maximum expected error of 0.5% and truncated at a length of 253 bp. Filtered reads maintained an average Phred Q score of 37.9. Chimeric sequences were identified and removed using the UCHIME algorithm with default settings^27^. A total of 172 out of 4,308 OTUs were removed after chimera checking and a total of 391,649 paired sequences were used in downstream analyses. OTUs were picked *de novo* using the UPARSE pipeline^28^ within USEARCHv7 using a 97% ID setting. Taxonomy was assigned using the assign_taxonomy.py script in QIIME 1.9.0^29^ with default parameters using the Greengenes 16S rRNA gene database (13-5 release, 97%)^30^. Results were compiled into a biological observation matrix (biom) format OTU table in which singleton sequences were removed.

### Analysis of the mycobiome

The ITS region between the 18S and 5.8S rRNA genes was amplified in 25 µL PCR reactions using the same concentrations as previously mentioned, with the exception of adding 5 µL of undiluted template DNA, and the use of ITS1 forward (5′-AATGATACGGCGACCACCGAGATCTACACGGCTTGGTCATTTAGAGGAAGTAA-3′) and Illumina-barcoded ITS2 reverse primers (5′-CAAGCAGAAGACGGCATACGAGAT TACCGCTTCTTC CG GCTGCGTTCTTCATCGATGC-3′) designed to avoid common PCR-related biases in generating fungal amplicons of the variable target region^31^. The protocol described by Smith and Peay is one of the only one-step PCR assays compatible with Illumina sequencing and ITS1F is highly fungal specific and other primers for ITS2 are often less specific and can co-amplify host tissue^32^. The thermocycling conditions performed using an MJ Research PTC-200 thermocycler (Bio-Rad, Hercules, CA) were: 94 °C for 1 min, 30 cycles of 94 °C for 30 s, 52 °C for 30 s, and 68 °C for 30 s; 68 °C for 7 min and a 4 °C hold. PCR products were visualized on a 1% SYBRsafe E-gel (Life Technologies, Carlsbad, CA). Purified libraries were pooled and sequenced on the Illumina NextSeq platform using the 150 bp paired end chemistry at University of California Davis Genome Center.

A total of 6,116,765 forward reads and 419,207 reverse reads were retrieved from ITS sequencing. Due to the higher read counts and the fact that we could retain more samples in our fungal dataset, only the forward reads were analyzed. This approach was used by Nguyen *et al*. who found that analyzing forward reads for ITS region 1 yielded a more robust analysis in a mock community analysis^33^. Forward ITS sequences were quality filtered using VSEARCH 1.11.1 with a maximum expected error of 0.5% and truncated at a length of 150 bp to retain an average Phred Q score of 35.6 throughout the entire read length. The sequences were trimmed using Trimmomatic to remove low-quality regions^32^. OTUs were picked using the open-reference UCLUST algorithm in QIIME 1.9.0^29^ at the default OTU threshold of 0.97 and singleton sequences were discarded. Taxonomy was assigned using the BLAST option in the assign_taxonomy.py script against version 7 of the UNITE fungal ITS database^34, 35^ with the maximum e-value set to the default of 0.001. Taxa with no BLAST hits were removed from the OTU table for downstream analysis.

### Diversity and Statistical Analyses

Alpha diversity rarefaction curves were created within the QIIME 1.9.1 package^29^ using the untransformed OTU table. Multiple rarefactions were performed on the 16S rRNA OTU table from all samples using a minimum depth of 0 sequences to a maximum depth of 7000 sequences, with a step size of 700 for 20 iterations. Multiple rarefactions were performed on the ITS OTU table from all samples using a minimum depth of 0 sequences to a maximum depth of 6000 sequences, with a step size of 600 for 20 iterations. Rarefactions were then collated and plotted using observed species, Chao1, PD Whole Tree, and Heip’s evenness diversity metrics. Alpha diversity was compared between disease states, as well as age, sex, BMI, smoking history, and antibiotic administration. Richness plots were made within Phyloseq using the untransformed OTU table against the Observed OTUs, Chao1, and ACE metrics with lines connecting each patient’s diverticular and normal samples.

Both 16S and ITS OTU tables were normalized using metagenomeSeq’s Cumulative Sum Scaling (CSS) algorithm^36^. Beta diversity analyses were performed using weighted UniFrac distance matrices and visualized using a 3-dimensional principal coordinate analysis (PCoA) plots in EMPeror. To assess within-group variation in DT and AT samples, average weighted UniFrac distances within each tissue grouping were calculated and compared using a two-sample *t*-test. Core microbiome analyses were also completed within QIIME 1.9.1^29^. The ANOSIM method within QIIME was employed on the weighted UniFrac distance matrices to test if there was a difference in beta diversity between DT and AT groups. LEfSe analysis was used to identify bacterial taxa whose sequences are differentially abundant between DT and AT groups^37^. LEfSe uses a Kruskal-Wallis test followed by pairwise Wilcoxon rank sum test correction, with both alpha values set to 0.05. The effect size cutoffs to determine significantly differentiating taxa between DT and AT groups were set at a genus-level LDA score >1.5 for both 16S rRNA gene and ITS data.

Co-occurrence networks were built and visualized in Cytoscape 3.3.0 using the CoNet plugin^38^. As created in QIIME 1.9.1, untransformed OTU tables consisting of exclusively chronically diseased tissue and adjacent tissue were uploaded. In a preprocessing step, any taxa appearing in <50% of samples were discarded and taxa had to appear as non-zero values in at least two samples to be considered for correlations to account for read count sparsity. Two correlational measures, Pearson and Spearman, and two dissimilarity measures, Bray-Curtis and Kullback-Leibler, were used to calculate correlations between the remaining taxa. The use of all four measures reduces the chance of spurious correlations due to outliers, matching zeroes, or data compositionality. The Benjamini-Hochberg-Yekutieli multiple testing correction was used to adjust *P*-values in the final step of CoNet processing.

Unassigned taxa were removed from networks. Remaining taxa were then labeled to the furthest identified taxonomic rank. Green lines were used to connect positive correlations, while red lines showed negative correlations. Chronically diseased tissue and adjacent tissue networks were additionally merged using both difference and intersection parameters. There were no shared correlations between DT and AT networks, suggesting differential correlations between bacteria and fungi in the different tissue types.

Predicted metagenomes were calculated with 16S rRNA gene data for the DT and AT sample groups using PICRUSt software^39^. A closed reference 16S rRNA gene OTU table was imported into PICRUSt version 1.1.0 mapped against the Kyoto Encyclopedia of Genes and Genomes (KEGG) database and summarized at the Level 3 functional annotation.

### Power analysis

The *micropower* R-package was used to calculate the PERMANOVA power of our study design^40^. The input for this analysis was the distance matrix made using weighted UniFrac distances from the 16S rRNA OTU table. PERMANOVA power was calculated with five, nine, and 15 subjects per treatment group specified. Each sample size was observed at alpha cutoffs of 0.01, 0.05, and 0.1 performed over 100 bootstrap iterations. The PERMANOVA power calculated from our study design of nine subjects per treatment group at an alpha cutoff of 0.05 was found to be 0.89 (Supplemental Table S1). Varying the alpha cutoffs to 0.01 and 0.1 changed our power calculations as described in Supplemental Table S1.

## Electronic supplementary material

Supplementary Information
