# Supplementary material for: The Microbial Ecosystem Distinguishes Chronically Diseased Tissue from Adjacent Tissue in the Sigmoid Colon of Chronic, Recurrent Diverticulitis Patients
Source: Sci Rep. 2017 Aug 16;7:8467. doi: 10.1038/s41598-017-06787-8 (PMC5559482; doi:10.1038/s41598-017-06787-8)
Supplement: Supplementary file 1 — Supplementary Information [file 41598_2017_6787_MOESM1_ESM.pdf]

The Microbial Ecosystem Distinguishes Chronically Diseased Tissue from Adjacent Tissue in the Sigmoid Colon of Chronic, Recurrent Diverticulitis Patients

Kathleen M. Schieffer[1,#], Kate Sabey[2,#], Justin R. Wright[2,3], David R. Toole[2], Rebecca Drucker[2], Vasily Tokarev[2], Leonard R. Harris[1], Sue Deiling[1], Melanie A. Eshelman[1,4], John P. Hegarty[1], Gregory S. Yochum[1,4], Walter A. Koltun[1], Regina Lamendella[2,3], David B. Stewart Sr.[1*]

Supplemental Table S1. PERMANOVA power analysis using the weighted UniFrac distances from our 16S rRNA OTU data table

Subjects/group	Alpha	Average effect size (omega2)	Power
5	0.01	0.059	0.1
9	0.01	0.055	0.4
15	0.01	0.057	1
5	0.05	0.056	0.24
9	0.05	0.059	0.89
15	0.05	0.058	1
5	0.1	0.058	0.5
9	0.1	0.061	0.95
15	0.1	0.059	1

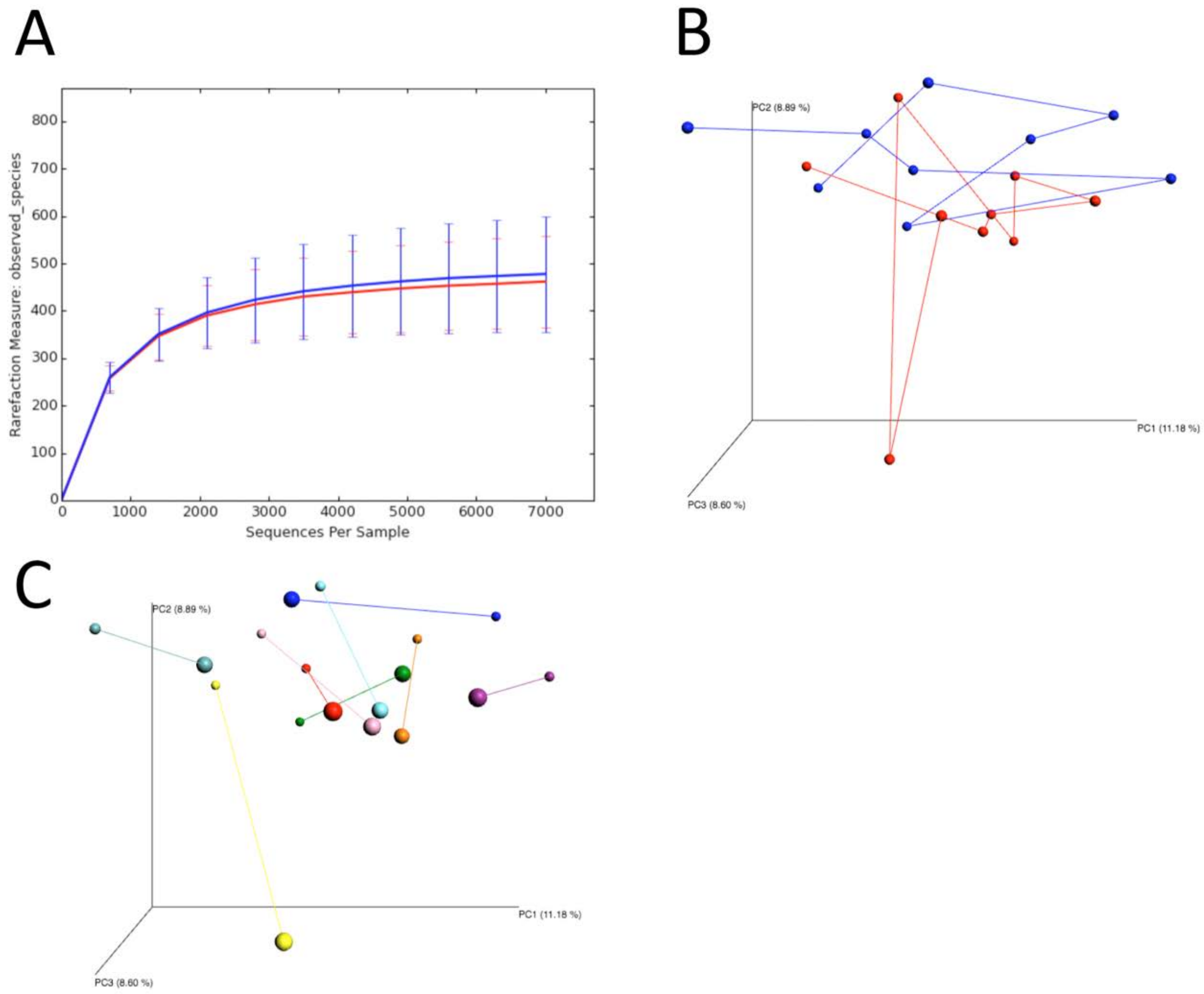

Supplemental Figure S1. Microbial alpha and beta diversity analysis. **(A)** Alpha diversity rarefaction plots of observed taxa within diverticulitis (red) tissue and adjacent (blue) tissue were generated within QIIME 1.9.0. Differences in alpha diversity were not found to be statistically significant. **(B)** Principle coordinate analysis plot calculated from weighted UniFrac distances comparing bacterial community structures between diverticulitis (red) tissue and adjacent (blue) tissue. **(C)** Principle coordinate analysis plot calculated from weighted UniFrac distances comparing bacterial community structures between individual patients. Each color is indicative of an individual patient, with large points representing diverticular tissue, and small points representing adjacent tissue. Clustering was not found to be statistically significant based on tissue type nor patient.

Supplemental Table S2. Differentially expressed bacterial taxa unique between diverticulitis tissue (DT) and adjacent tissue (AT). Only taxonomically assigned OTUs at the kingdom are listed.

DT Core (80%) Bacteria	Phylum	Class	Order	Family	Genus	Species
	Proteobacteria	*Gammaproteobacteria*	*Enterobacteriales*	*Enterobacteriaceae*		
	Firmicutes	*Clostridia*	*Clostridiales*	*Peptococcaceae*	*Desulfosporosinus*	*meridiei*
	Firmicutes	*Clostridia*	*Clostridiales*	*Syntrophomonadaceae*	*Syntrophomonas*	
	Proteobacteria	*Gammaproteobacteria*	*Alteromonadales*	*Shewanellaceae*	*Shewanella*	
	Proteobacteria	*Alphaproteobacteria*	*Rhizobiales*	*Bradyrhizobiaceae*		
	Proteobacteria	*Betaproteobacteria*	*Burkholderiales*	*Oxalobacteraceae*		
	Proteobacteria	*Betaproteobacteria*	*Burkholderiales*	*Oxalobacteraceae*		
	Proteobacteria	*Betaproteobacteria*	*Burkholderiales*	*Comamonadaceae*		
	Proteobacteria	*Betaproteobacteria*	*Burkholderiales*	*Comamonadaceae*		
	Proteobacteria	*Betaproteobacteria*	*Burkholderiales*	*Comamonadaceae*		
	Proteobacteria	*Gammaproteobacteria*	*Pseudomonadales*	*Pseudomonadaceae*	*Pseudomonas*	*veronii*
	Proteobacteria	*Betaproteobacteria*	*Burkholderiales*	*Comamonadaceae*		
	Synergistetes	*Synergistia*	*Synergistales*	*Synergistaceae*	*vadinCA02*	
	Proteobacteria	*Gammaproteobacteria*	*Methylococcales*	*Methylococcaceae*		
	Actinobacteria	*Actinobacteria*	*Actinomycetales*	*Microbacteriaceae*		
	Proteobacteria	*Deltaproteobacteria*	*Desulfobacterales*	*Desulfobulbaceae*		
	Proteobacteria	*Alphaproteobacteria*	*Caulobacterales*	*Caulobacteraceae*		
	Proteobacteria	*Alphaproteobacteria*	*Sphingomonadales*	*Sphingomonadaceae*	*Sphingomonas*	
	Proteobacteria	*Alphaproteobacteria*	*Caulobacterales*	*Caulobacteraceae*		
	Firmicutes	*Bacilli*	*Bacillales*	*Bacillaceae*	*Bacillus*	*foraminis*

AT Core (80%) Bacteria	Phylum	Class	Order	Family	Genus	Species
	Bacteroidetes	*Cytophagia*	*Cytophagales*	*Cytophagaceae*	*Hymenobacter*	
	GN04					
	Proteobacteria	*Betaproteobacteria*	*Burkholderiales*	*Comamonadaceae*		
	Proteobacteria	*Epsilonproteobacteria*	*Campylobacterales*	*Campylobacteraceae*	*Arcobacter*	
	Bacteroidetes	*Sphingobacteriia*	*Sphingobacteriales*			
	Proteobacteria	*Epsilonproteobacteria*	*Campylobacterales*	*Helicobacteraceae*		
	Bacteroidetes	*Bacteroidia*	*Bacteroidales*			
	Proteobacteria	*Betaproteobacteria*	*Methylophilales*	*Methylophilaceae*	*Methylotenera*	*mobilis*
	Cyanobacteria	*Chloroplast*	*Stramenopiles*			
	Proteobacteria	*Deltaproteobacteria*	*Desulfuromonadales*	*Geobacteraceae*	*Geobacter*	
	Chloroflexi	*Anaerolineae*	*H39*			
	Bacteroidetes	*Flavobacteriia*	*Flavobacteriales*	*Cryomorphaceae*	*Fluviicola*	
	Bacteroidetes	*Flavobacteriia*	*Flavobacteriales*	*Cryomorphaceae*	*Fluviicola*	
	Acidobacteria	*[Chloracidobacteria]*	*Ellin7246*			
	Proteobacteria	*Betaproteobacteria*	*Burkholderiales*	*Comamonadaceae*	*Rhodoferax*	

Supplemental Table S3. Enriched bacterial and archaeal taxa identified by LefSe analysis.

	Enriched Bacterial and Archaeal Taxonomy	LDA Score	LefSe *P* value
DT	*Archaea.Crenarchaeota.Thaumarchaeota*	3.22	0.007
	Archaea.Crenarchaeota.Thaumarchaeota.Cenarchaeales	2.87	0.031
	Archaea.Crenarchaeota.Thaumarchaeota.Cenarchaeales.Cenarchaeaceae	2.73	0.039
	Archaea.Euryarchaeota.Thermoplasmata	2.82	0.029
	Bacteria.Acidobacteria.Acidobacteria_6	3.07	0.038
	Bacteria.Actinobacteria.Acidimicrobiia.Acidimicrobiales	3.15	0.020
	Bacteria.Actinobacteria.Actinobacteria.Actinomycetales.Microbacteriaceae	3.42	0.019
	Bacteria.Proteobacteria.Alphaproteobacteria.Rhodeobacterales.Rhodobacteraceae.Pseudoruegeria	2.84	0.031
	Bacteria.Proteobacteria.Betaproteobacteria_SC_I_84	2.89	0.029
	Bacteria.Proteobacteria.Deltaproteobacteria.Desulfobacterales.Desulfobulbaceae	3.03	0.024
	Bacteria.Spirochaetes.Leptospirae.Leptospirales_Sediment_4_SJA_88	2.88	0.029
AT	*Bacteria.Actinobacteria.Actinobacteria.Actinomycetales.Kineosporiaceae*	2.87	0.031
	Bacteria.Actinobacteria.Thermoleophilia	3.18	0.038
	Bacteria.Actinobacteria.Thermoleophilia.Gaiellales.Gaiellaceae	2.99	0.007
	Bacteria.Armatimonadetes_0319_6E2	2.97	0.012
	Bacteria.Chloroflexi.Anaerolineae_H39	2.92	0.047
	Bacteria.Cyanobacteria_4C0d_2_MLE1_12	2.85	0.029
	Bacteria.Gemmatimonadetes_Gemm_5	2.75	0.029
	Bacteria.Planctomycetes_OM190	2.97	0.024
	Bacteria.Planctomycetes.Phycisphaerae.Phycisphaerales	2.75	0.029
	Bacteria.Proteobacteria.Alphaproteobacteria.Rhizobiales.Hyphomicrobiaceae.Parvibaculum	2.85	0.029
	Bacteria.Proteobacteria.Alphaproteobacteria.Sphingomonadales	2.89	0.018
	Bacteria.Proteobacteria.Deltaproteobacteria.Desulfovibrionales.Desulfovibrionaceae	2.75	0.012
	Bacteria.Proteobacteria.Gammaproteobacteria.Alteromonadales.Alteromonadaceae.Marinobacter	2.91	0.008
	Bacteria.Proteobacteria.Gammaproteobacteria.Pseudomonadales.Pseudomonadaceae.Pseudomonas	3.31	0.038

Supplemental Table S4. Enriched predicted function from PICRUSt analysis.

	**Enriched Function**	**LDA Score**	**LefSe *P* value**
DT	C5 Branched dibasic acid metabolism	1.60	0.009
	Methane metabolism	2.30	0.047
	Valine, leucine, and isoleucine biosynthesis	1.85	0.031
AT	Carbohydrate metabolism	1.58	0.024
	Glycosyltransferases	1.85	0.002

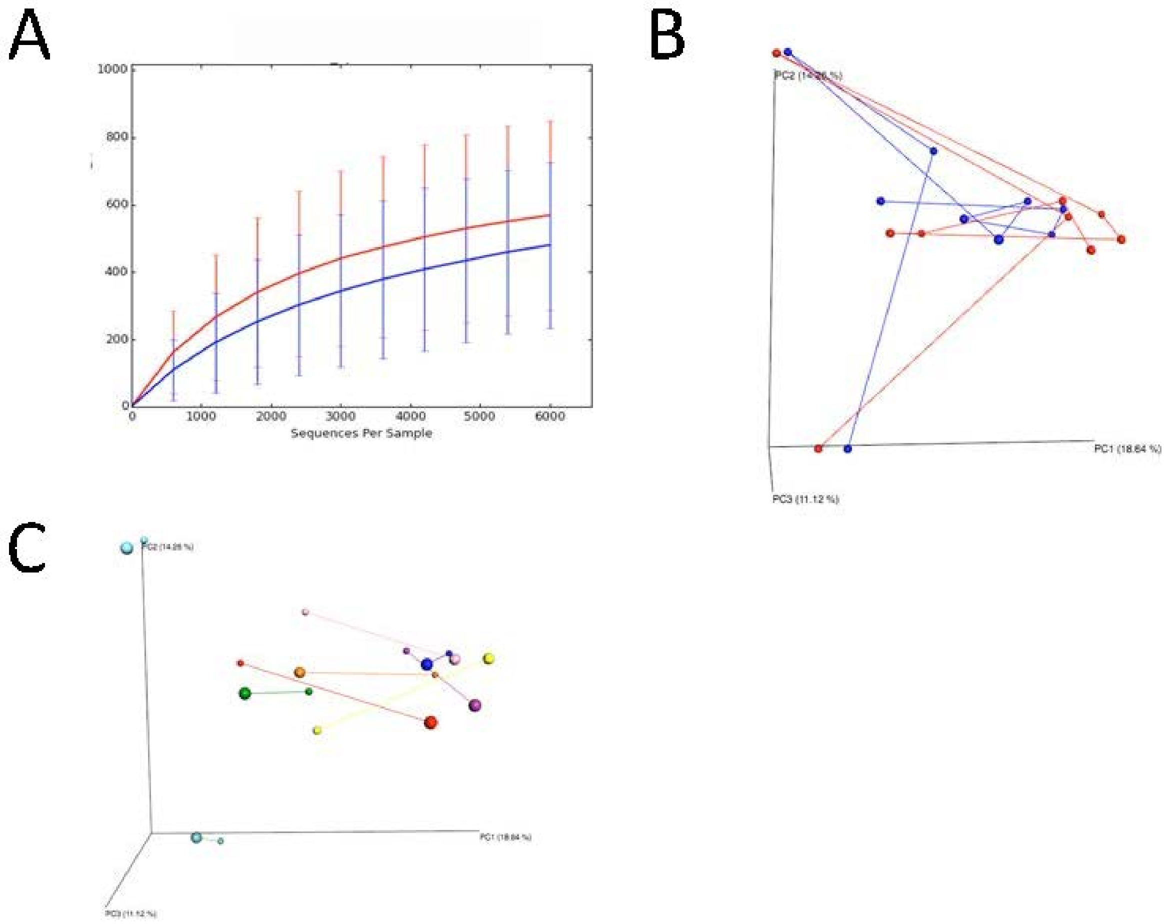

Supplemental Figure S2. Fungal alpha and beta diversity analysis. **(A)** Alpha diversity rarefaction plots of observed taxa within diverticulitis (red) tissue and adjacent (blue) tissue were generated within QIIME 1.9.0. Differences in alpha diversity were not found to be statistically significant. **(B)** Principle coordinate analysis plot calculated from weighted UniFrac distances comparing bacterial community structures between diverticulitis (red) tissue and adjacent (blue) tissue. **(C)** Principle coordinate analysis plot calculated from weighted UniFrac distances comparing bacterial community structures between individual patients. Each color is indicative of an individual patient, with large points representing diverticular tissue, and small points representing adjacent tissue. Clustering was not found to be statistically significant based on tissue type, but was found to be statistically significant based on patient ID (ANOSIM *P*=0.01).

Supplemental Table S5. Differentially expressed fungal taxa unique between diverticulitis tissue (DT) and adjacent tissue (AT). Only taxonomically assigned OTUs at the kingdom are listed.

DT Core (80%)<br>Fungi	Phylum	Class	Order	Family	Genus	Species
	Ascomycota	*Leotiomycetes*				

AT Core (80%)<br>Fungi	Phylum	Class	Order	Family	Genus	Species
	Ascomycota	*Saccharomycetes*	*Saccharomycetales*	*Incertae sedis*	*Cyberlindnera*	*jadinii*
	Ascomycota	*Eurotiomycetes*	*Eurotiales*	*Trichocomaceae*	*Aspergillus*	*subversicolor*

**Supplemental Table S6. Enriched fungal taxonomy from LefSe analysis.**			
	**Enriched function**	**LDA score**	**LefSe *P* value**
DT	*Fungi.Ascomycota.Chaetothyriomycetes.Chaetothyriales.Herpotrichiellaceae.Exophiala*	4.11	0.037
AT	*Fungi.Basidiomycota.Agaricomycetes. Agaricomycetidae.Agaricales*	4.04	0.039
	Fungi.Basidiomycota.Agaricomycetes.Agaricomycetidae.Agaricales.Plutaceae	4.24	0.039
	Fungi.Basidiomycota.Agaricomycetes.Agaricomycetidae.Agaricales.Plutaceae.Pluteus	4.21	0.039
